# Age structure in SIRD models for the COVID-19 pandemic—A case study on Italy data and effects on mortality

**DOI:** 10.1371/journal.pone.0264324

**Published:** 2022-02-24

**Authors:** Giuseppe Carlo Calafiore, Giulia Fracastoro

**Affiliations:** Department of Electronics and Telecommunications, Politecnico di Torino, Torino, Italy; Texas A&M University College Station, UNITED STATES

## Abstract

The COVID-19 pandemic is bringing disruptive effects on the healthcare systems, economy and social life of countries all over the world. Even though the elder portion of the population is the most severely affected by the COVID-19 disease, the counter-measures introduced so far by governments took into little account the age structure, with restrictions that act uniformly on the population irrespectively of age. In this paper, we introduce a SIRD model with age classes for studying the impact on the epidemic evolution of lockdown policies applied heterogeneously on the different age groups of the population. The proposed model is then applied to age-stratified COVID-19 Italian data. The simulation results suggest that control measures focused to specific age groups may bring benefits in terms of reduction of the overall mortality rate.

## Introduction

**Fig 1:**
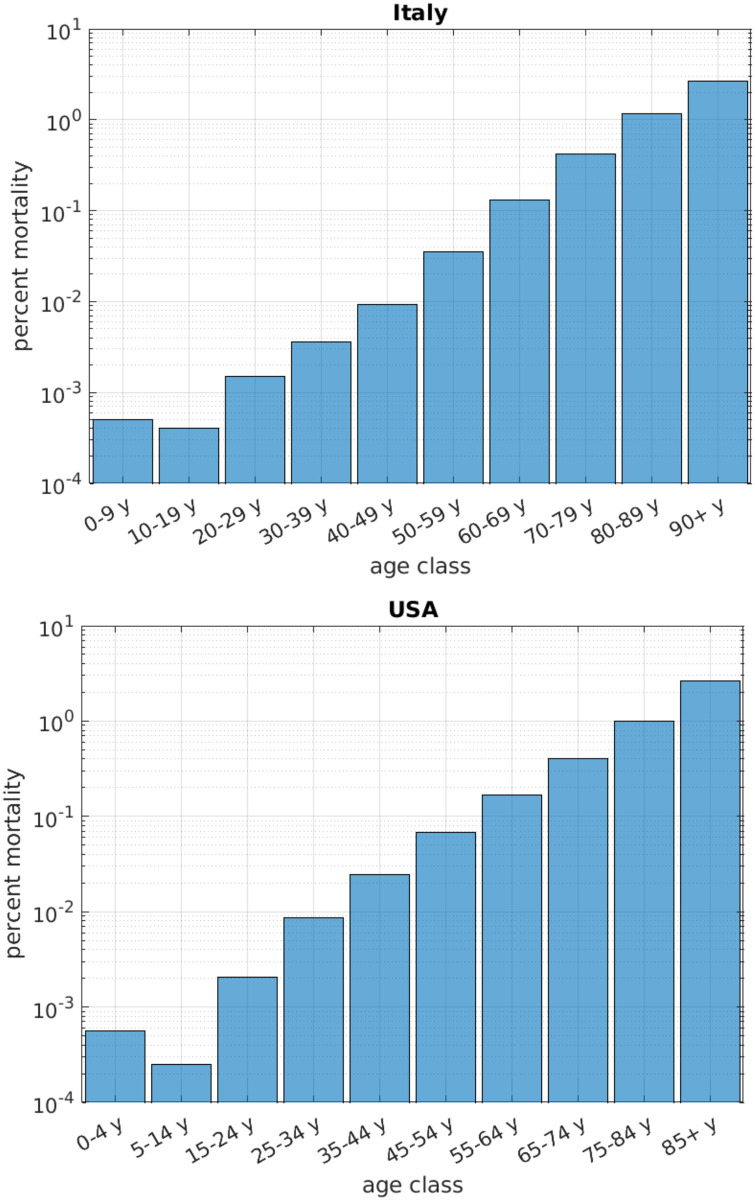
Mortality of the COVID-19 disease per age class. Italy data (top) based on COVID-19 data from [11]; US data (bottom) based on COVID-19 data from https://data.cdc.gov/NCHS/Provisional-COVID-19-Death-Counts-by-Sex-Age-and-S/9bhg-hcku, updated May 19, 2021, and on demographics data from https://www.statista.com/statistics/241488/population-of-the-us-by-sex-and-age.

Governments across the globe are struggling to face the global COVID-19 pandemic, enacting rules aimed at limiting the spread of the contagion and at safeguarding the capacity of the healthcare systems, ultimately protecting the population from the most adverse outcomes of the disease. To date (May 2021) the COVID-19 disease has produced a total of about 165 million cases worldwide, and 3.5 million deaths [1]. The United States has been one of the most severely hit countries, with a total of over 33 million cases and 6 hundred thousand deaths to date [2]. The counter-measures enforced for controlling the contagion have been of diverse intensity in different countries, ranging from bland (e.g., in Sweden) to medium (e.g., USA) and strong (e.g., Italy and China) [3–5]. In all cases, the control measures included bans of various degree in personal mobility and travel, closures of commercial activities, bars, shops and restaurants, interdiction of gathering in public places such as parks and beaches, closure of schools and, in extreme cases, the shut down of industrial activities. One common aspect of these restrictions, however, was that they acted over the population irrespective of age. This somehow contrasts with the fact that the effects of the COVID-19 disease appear to have increasing severity with the age of the infected individuals, the elderly unfortunately accounting for a large portion of the fatal cases [6–8]. This heterogeneity in the age distribution of mortality has been observed in many countries, with similar trends [9, 10]. Fig 1, for instance, shows the percent mortality from the COVID-19 disease in Italy and in the US, in logarithmic scale, as a function of the age class of the population: the increase of mortality with age is exponential, in particular the mortality of the elder class of individuals aged 85 or more is 300 times higher than that of individuals under 30 years old.

Clearly, in principle, heterogeneity in the mortality distribution can be attributed to (i) the age dependency in susceptibility to the infection and/or to (ii) the age dependency of the severity of the symptoms and outcome of the disease probably due to a weaker immune system or the presence of comorbidities. A recent study [12] on COVID-19 cases in Italy, Japan and Spain, however, indicates that the contribution of age-dependency to susceptibility is not supported by existing data, while the age-dependencies of the mortality rate seems to determine the age distribution in mortality from COVID-19.

The situation in most countries is thus such that on the one hand the part of the population of schooling age and of working age is the most strongly impacted by the governments’ restrictions, and, on the other hand, the same portion of the population is the least affected by the infection, at least in terms of mortality. The importance of the population age structure in determining the pandemic’s progression and impact has been well recognized by researchers, see, e.g., [6]. Indeed, demographic science shows how the effects of the pandemic can be dramatically different in populations with similar sizes but different age structures. Despite this evidence, however, governments have so far largely neglected age structure in the definition of their non-pharmaceutical policies against the pandemic. Even data on case and fatality disaggregated by age is scarcely available to researchers, and calls for countries to provide this data have been repeatedly made, see, e.g., [6, 13]. Also, in a quite debated and controversial declaration [14], a group of renowned scientists claimed that current lockdown policies are producing devastating effects on short and long-term public health and, recognizing that vulnerability to death from COVID-19 is very different in young-aged subjects than it is in the elder, proposed a “focused protection” approach, whose philosophy would be “to allow those who are at minimal risk of death to live their lives normally to build up immunity to the virus through natural infection, while better protecting those who are at highest risk.”

The main scope of this paper is to explore via an analytical model the effects on the contagion evolution of control policies that act heterogeneously on the different age groups of the population. Since age is an important risk factor for severe COVID-19 symptoms, this can be an additional control strategy to be considered for reducing the impact of the epidemic. Other different control strategies have already been proposed, such as quarantine and strict isolation policies [15, 16], regional strategies [17–19] or intermittent lockdowns [20].

In this paper, we first propose a modified Susceptible-Infectious-Recovered-Deceased (SIRD) model with age classes for describing the mean-field time evolution of the number of susceptible, infected, recovered and deceased individuals in each of the considered age groups. Next, we pose this model in a suitable regression form which is amenable to an efficient numerical scheme for the identification of the model parameters from real observed data. This model is then trained on age-grouped COVID-19 Italian data covering the period from March 1st, 2020 to March 20th, 2021, [11]. This constitutes the reference baseline for evaluating the effects of additional control measures. Control policies applied heterogeneously on the age classes are next introduced in the model, tuning the estimated transmission rate parameters of specific age groups. In particular, two different scenarios are investigated. The first one considers a strengthened lockdown applied to the eldest portion of the population. Instead, the second scenario considers a relaxation of the restrictions for the population of schooling age. This second scenario is useful for investigating the effects of the school openings.

The results of these numerical experiments suggest that selective restrictions applied only on the eldest age classes can significantly reduce the spread of the epidemic, while relaxing the restrictions of the youngest age classes yields a negligible variation of the overall mortality rate.

## A modified SIRD model with age classes

We consider a geographical region, assumed as isolated from other regions, and within such region we let the population be divided in *K* non-overlapping age classes. For each class *i*, *i* = 1, …, *K*, we define the following quantities:

- *S*_*i*_(*t*): the number of individuals in the *i*th class *susceptible* of contracting the infection at day *t*;
- *I*_*i*_(*t*): the number of individuals in the *i*th class that are alive and *infected* at day *t*;
- *R*_*i*_(*t*): the cumulative number of individuals in the *i*th class that *recovered* from the disease up to day *t*;
- *D*_*i*_(*t*): the cumulative number of individuals in the *i*th class that are *deceased* due to the disease, up to day *t*.

The following assumptions underpin the model we develop: (a) individuals do not change age class as *t* evolves, that is the age class is assigned at time *t* = 0 and then remains fixed for the time range of our study; (b) the region under consideration is assumed to be isolated from other regions, so that the total population remains constant in size; (c) the recovered subjects are no longer susceptible of infection, at least within the time range of our study; (d) deaths due to other reasons (different from the disease under consideration) are neglected. We let

$$C_{i}\left(t\right)≐S_{i}\left(t\right)+I_{i}\left(t\right)+R_{i}\left(t\right),\;i=1,\ldots ,K$$

denote the total number of individuals in the *i*th class, and

$$C_{i}\left(t\right)≐\sum_{i=1}^{K}C_{i}\left(t\right)=S\left(t\right)+I\left(t\right)+R\left(t\right)$$

denote the total number of individuals in the population, being

$$S\left(t\right)≐\sum_{i=1}^{K}S_{i}\left(t\right),\;I\left(t\right)≐\sum_{i=1}^{K}I_{i}\left(t\right),\;R\left(t\right)≐\sum_{i=1}^{K}R_{i}\left(t\right).$$

We denote with $S_{i}\left(t\right)$ the set of susceptible individuals in class *i* on day *t*, and with $I_{i}\left(t\right)$ the set of infected individuals in class *i* on day *t*, for *i* = 1, …, *K*. For each class pair (*i*, *j*), we denote with *c*_*ij*_(*t*) the average number of class-*j* individuals with whom an individual of class *i* comes into contact during day *t*. The contact factor *c*_*ij*_ depends on the size of the class-*j* population, and on the behavior and activity levels of the classes, which may also reflect regulatory interventions such as mobility reductions and lock-downs. A fraction *S*_*j*_(*t*)/*C*_*j*_(*t*) of the *c*_*ij*_(*t*) contacts involve susceptible individuals, and in such cases an actual contagion occurs with probability *ξ*, where *ξ* denotes the probability of infection transmission of the virus. Overall, we obtain the following expression for the average number of infections that an individual in $I_{i}\left(t\right)$ produces on individuals in $S_{j}\left(t\right)$:

$$n_{ij}\left(t\right)=\xi c_{ij}\left(t\right)\frac{S_{j}\left(t\right)}{C_{j}\left(t\right)}=\beta_{ij}\left(t\right)\frac{S_{j}\left(t\right)}{C_{j}\left(t\right)},$$

where we defined the *transmission-rate* parameter from the *i*th class to the *j*th class as

$$\beta_{ij}\left(t\right)≐\xi \gamma_{ij}\left(t\right).$$

The ensemble of the *I*_*i*_(*t*) individuals will cause, on average, *N*_*ij*_(*t*) new infections in class *i* on day *t*, where

$$N_{ij}\left(t\right)≐\beta_{ij}\left(t\right)I_{i}\left(t\right)\frac{S_{j}\left(t\right)}{C_{j}\left(t\right)}.$$

Overall, the average number of new daily contagions in class *j* generated collectively by the infected individuals of all classes during day *t* is

$$\begin{array}{c} N_{j}\left(t\right)≐\sum_{i=1}^{K}N_{ij}\left(t\right)=\sum_{i=1}^{K}\beta_{ij}\left(t\right)I_{i}\left(t\right)\frac{S_{j}\left(t\right)}{C_{j}\left(t\right)}. \end{array}$$ (1)

**Remark 1 (Model parsimony)** We observe that, in practice, the transmission-rate parameters *β*_*ij*_(*t*) appearing in Eq (1) are unknown. It is actually one of the goals of the present work to propose an effective technique for estimating these parameters (together with the other model parameters discussed further on) by leveraging the available information from the system, that is the measurements of the daily infected, recovered and deceased individuals. For a model with, say, *K* = 10 age classes, such as the one considered in the experiments presented in this paper, at each *t* one would have *K*^2^ = 100 transmission parameters to be estimated on the basis of few measurements (precisely, 3*K* measurements at each *t*). Even though the model parameters are encouraged to have few variations over the estimation horizon (we discuss this when we present the model with time-varying parameters, say, *αT* variations, where *T* is the number of days for which the data is available and *α* ≤ 1, we would have *αTK*^2^ transmission parameters to estimate on the basis of 3*TK* measurements, and it is apparent that the number of parameters grows faster than the number of available measurements. It is well known in the literature (see, e.g., an early reference in [21]) that over-parameterized models tend to overfit the data and to provide unstable and noisy estimates of the parameters.

For the these reasons, we purposely employ in the remainder of this paper an explanatory model for the observed data which uses a simplified version of Eq (1). Precisely, we shall assume that, for each class *j*, it holds that *β*_*ij*_(*t*) = *β*_*j*_(*t*) for all *i*. In words, the transmission rates from class *i* to class *j* depend only on the receiver class *j*. With such position, Eq (1) is rewritten as

$$\begin{array}{c} N_{j}\left(t\right)=\beta_{j}\left(t\right)I\left(t\right)\frac{S_{j}\left(t\right)}{C_{j}\left(t\right)}, \end{array}$$ (2)

and the total number of transmission parameters to be estimated is reduced to a more manageable number of *αTK*.

Next, we consider that during day *t* a fraction *γ*_*i*_(*t*) of the infected individuals in class *i* recovers, and a fraction *ν*_*i*_(*t*) of them dies from the disease. The above setup, using the simplified Eq (2), leads to the formulation of the following discrete-time dynamic equations for the evolution of the contagion: for *i* = 1, …, *K* and *t* = 0, 1, …,

$$S_{i}\left(t\;+\;1\;\right)\;=\;S_{i}\left(t\right)-\beta_{i}\left(t\right)I\left(t\right)\frac{S_{i}\left(t\right)}{C_{i}\left(t\right)}$$ (3)

$$\;I_{i}\left(t\;+\;1\;\right)\;=\;I_{i}\left(t\right)\;+\;\beta_{i}\left(t\right)I\left(t\right)\frac{S_{i}\left(t\right)}{C_{i}\left(t\right)}\;-\;\gamma_{i}\left(t\right)I_{i}\left(t\right)\;-\;\nu_{i}\left(t\right)I_{i}\left(t\right)$$ (4)

$$\;R_{i}\left(t\;+\;1\;\right)\;=\;R_{i}\left(t\right)+\gamma_{i}\left(t\right)I_{i}\left(t\right)$$ (5)

$$\;D_{i}\left(t\;+\;1\;\right)\;=\;D_{i}\left(t\right)+\nu_{i}\left(t\right)I_{i}\left(t\right).$$ (6)

The model is initialized at some conventional *t* = 0 with values *S*_*i*_(0)>0, *I*_*i*_(0)>0, *R*_*i*_(0)≥0, and *D*_*i*_(0) = 0, for *i* = 1, …, *K*. Notice that it holds for all *i* = 1, …, *K* that

$$\begin{array}{cc} & S_{i}\left(t+1\right)+I_{i}\left(t+1\right)+R_{i}\left(t+1\right)+D_{i}\left(t+1\right)\\ & =S_{i}\left(t\right)+I_{i}\left(t\right)+R_{i}\left(t\right)+D_{i}\left(t\right)=P_{i},\;\forall t, \end{array}$$

where *P*_*i*_ = *S*_*i*_(0) + *I*_*i*_(0) + *R*_*i*_(0) + *D*_*i*_(0) is the initial total population for the *i*th class. The *i*th class population *P*_*i*_ is assumed to be a fixed fraction *α*_*i*_ of the total population *P* exposed to the contagion. The fractions *α*_*i*_, *i* = 1, …, *K*, are obtained from demographic data. The total exposed population *P* is in turn assumed to be only a portion of the actual population *Pop* of the region of interest. Denoting by *ω* ∈ [0, 1] the (unknown) coefficient of proportionality in *P* = *ωPop*, we have that

$$S_{i}\left(t\right)+I_{i}\left(t\right)+R_{i}\left(t\right)+D_{i}\left(t\right)=P_{i}=\alpha_{i}P=\alpha_{i}\omega \text{Pop},\;\;\forall t\geq 0,$$

where *α*_*i*_ is given, while *ω* is one of the model parameters to be estimated from the observed data. The last equation is used in the identification phase for obtaining the number of susceptible individuals in the *i*th class, since this number is not directly measurable otherwise:

$$\begin{array}{c} S_{i}\left(t\right)=\alpha_{i}\omega \text{Pop}-I_{i}\left(t\right)-R_{i}\left(t\right)-D_{i}\left(t\right). \end{array}$$ (7)

## Model identification

We first consider a constant-parameter version of the model; then we introduce the time varying extension.

### Regression model with constant parameters

If *β*_*j*_(*t*) = *β*_*j*_, *γ*_*j*_(*t*) = *γ*_*j*_, and *ν*_*j*_(*t*) = *ν*_*j*_ for all *t* and all *j* = 1, …, *K*, the model (3)–(6) can be rewritten in the following regression form, for *i* = 1, …, *K* and *t* = 0, 1, …

$$\begin{array}{c} \Delta_{i}\left(t+1\right)=\Phi_{i}\left(t,\omega \right)\theta , \end{array}$$ (8)

where

$$\begin{array}{cc} \Phi_{i}\left(t,\omega \right) & ≐[\begin{array}{ccc} -\frac{S_{i}\left(t\right)}{C_{i}\left(t\right)}I\left(t\right)e_{i}^{⊤} & 0_{K}^{⊤} & 0_{K}^{⊤}\\ \frac{S_{i}\left(t\right)}{C_{i}\left(t\right)}I\left(t\right)e_{i}^{⊤} & -I_{i}\left(t\right)e_{i}^{⊤} & -I_{i}\left(t\right)e_{i}^{⊤}\\ 0_{K}^{⊤} & I_{i}\left(t\right)e_{i}^{⊤} & 0_{K}^{⊤}\\ 0_{K}^{⊤} & 0_{K}^{⊤} & I_{i}\left(t\right)e_{i}^{⊤} \end{array}],\\ \theta & ≐{\left[\begin{array}{c} \beta \;\gamma \;\nu \end{array}\right]}^{⊤}, \end{array}$$

**0**_*K*_ is a vector of zeros of dimension *K*, ***e***_*i*_ is a vector of dimension *K* with a one in position *i* and zeros elsewhere, ***β***^⊤^ ≐ [*β*_1_⋯*β*_*K*_], ***γ***^⊤^ ≐ [*γ*_1_⋯*γ*_*K*_], ***ν***^⊤^ ≐ [*ν*_1_⋯*ν*_*K*_], and

$$\Delta_{i}\left(t+1\right)≐[\begin{array}{c} S_{i}\left(t+1\right)-S_{i}\left(t\right)\\ I_{i}\left(t+1\right)-I_{i}\left(t\right)\\ R_{i}\left(t+1\right)-R_{i}\left(t\right)\\ D_{i}\left(t+1\right)-D_{i}\left(t\right) \end{array}].$$

Our objective is to identify the model parameters *ω* ∈ [0, 1] and ***θ*** ≥ 0 on the basis of observed data. For a given time horizon *T* > 0, the observed data at *t* = 0, 1, …, *T*, are *I*_*i*_(*t*), *R*_*i*_(*t*), *D*_*i*_(*t*) for each class *i* = 1, …, *K*. From these data, and for given *ω*, we construct *S*_*i*_(*t*) according to (7). Notice that the transition matrix **Φ**_*i*_(*t*, *ω*) depends on *ω* nonlinearly, through the dependence of *S*_*i*_(*t*) on *ω*.

We next define a quadratic cost with forgetting factor *w* ∈ (0, 1]

$$\begin{array}{c} f\left(\omega ,\theta \right)≐\frac{1}{T}\sum_{t=0}^{T-1}w^{T-t}\sum_{i=1}^{K}{∥\Delta_{i}\left(t+1\right)-W\Phi_{i}\left(t,\omega \right)\theta ∥}_{2}^{2}, \end{array}$$ (9)

where *W* is a diagonal weight matrix, which takes into account the fact that the elements of **Δ**_*i*_(*t* + 1) might have different orders of magnitude.

The estimation problem amounts to solving min_*ω*,***θ***_ *f*(*ω*, ***θ***) under constraints that ***θ*** ≥ 0, *ω* ∈ [0, 1], and that *S*_*i*_(*t*)≥0 for all *t* = 0, 1, …, *T* and *i* = 1, …, *K*. These latter constraints are guaranteed to hold if

$$\omega \geq \omega_{\text{min}}≐\underset{i=1,\ldots ,K}{\text{max}}\underset{t=0,\ldots ,T}{\text{max}}\frac{I_{i}\left(t\right)+R_{i}\left(t\right)+D_{i}\left(t\right)}{\alpha_{i}\text{Pop}}.$$

We observe that, for fixed *ω*, the minimization of *f* with respect to ***θ*** = (***β***, ***γ***, ***ν***) can be done efficiently by solving a linearly constrained least-squares problem. We call this the inner step of the identification algorithm. The dependency of *f* on *ω* is instead non-convex, hence we approach this issue via an outer gridding on *ω* ∈ [*ω*_min_, 1], as detailed in the following algorithm.

**Algorithm 1** (**Estimation of constant parameters**)

1. Grid *n* values *ω*_*i*_ of *ω* in [*ω*_min_, 1]. For each of these *ω*_*i*_:
2. Solve the constrained least-squares problem $f_{i}^{*}={\text{min}}_{\theta \geq 0}f\left(\omega_{i};\theta \right)$ and let $\theta_{i}^{*}$ be an optimal solution.
3. At the end of the loop, retain the *ω*_*i*_ value that yielded the minimal value of $f_{i}^{*}$, and return this *ω*_*i*_ along with the corresponding $\theta_{i}^{*}$.

### Model with time-varying parameters

While a constant-parameters model may be appropriate for describing a specific phase in the evolution of a pandemic, it can hardly capture its overall characteristics over an extended period of time. Clearly, the contagion rates *β*_*i*_(*t*) vary due to changes in the behavior of the population, induced, for instance, by restrictive measures on people’s mobility imposed by authorities. Similarly, the recovery and death rates *γ*_*i*_(*t*), *ν*_*i*_(*t*) may change due to medical response policies that improve as knowledge and understanding of the virus progresses.

To incorporate time-varying coefficients into a tractable model, we consider next an approach with piece-wise constant parameters. That is, we let the parameters *β*_*j*_(*t*), *γ*_*j*_(*t*), *ν*_*j*_(*t*) be variable with time, but we impose appropriate restrictions so to insure that they remain constant over reasonable intervals of time, and only change value few times over the identification horizon [0, *T*]. Such model captures the fact that the parameters may vary with time while avoiding the overfitting that occurs if the variability is left unconstrained. Further, the identified periods over which the parameters are constant shall pinpoint the instants at which the changes of phase in the contagion happen, such as, e.g., the instant when lockdown measures are imposed or released.

The approach we propose is non-parametric: we let the values of *β*_*j*_(*t*), *γ*_*j*_(*t*), *ν*_*j*_(*t*) be variables in the optimization problem, at each *t* = 0, …, *T*, and we impose *sparsity* over the discrete-time derivative of these signals. That is, we impose that the discrete-time derivative of the parameter signals is zero everywhere except for a few values (which will be determined by the optimization algorithm), and this will imply that the parameter signals themselves are piece-wise constant, see for instance Section 9.5 of [22].

Recalling the model expression in (8), we modify it to

$$\begin{array}{c} \Delta_{i}\left(t+1\right)=\Phi_{i}\left(t,\omega \right)\theta \left(t\right), \end{array}$$ (10)

where all terms are defined as in the previous section, except for ***θ***(***t***) which now contains the time varying parameters:

$$\begin{array}{cc} \theta (t) & ≐{\left[\begin{array}{c} \beta^{⊤}\left(t\right)\;\gamma^{⊤}\left(t\right)\;\nu^{⊤}\left(t\right) \end{array}\right]}^{⊤}. \end{array}$$

Letting

$$\bar{\beta }≐[\begin{array}{c} \beta (0)\\ \beta (1)\\ \vdots \\ \beta (T) \end{array}],\;\bar{\gamma }≐[\begin{array}{c} \gamma (0)\\ \gamma (1)\\ \vdots \\ \gamma (T) \end{array}],\;\bar{\nu }≐[\begin{array}{c} \nu (0)\\ \nu (1)\\ \vdots \\ \nu (T) \end{array}],\;$$

We write the discrete-time derivative vectors as

$$\delta \bar{\beta }≐D\bar{\beta },\;\delta \bar{\gamma }≐D\bar{\gamma },\;\delta \bar{\nu }≐D\bar{\nu },$$

where

$$D≐[\begin{array}{cccccc} -I_{K} & I_{K} & 0 & 0 & \cdots & 0\\ 0 & -I_{K} & I_{K} & 0 & \cdots & 0\\ \vdots & \vdots & \vdots & \vdots & \cdots & \vdots \\ 0 & 0 & \cdots & 0 & -I_{K} & I_{K} \end{array}]$$

is a block matrix of size *TK* × (*T* + 1)*K*. Let further

$$E_{k}≐\text{blockdiag}(e_{k}^{⊤},e_{k}^{⊤},\ldots ,e_{k}^{⊤})\in R^{T,KT},\;k=1,\ldots ,K,$$

where *e*_*k*_ is a vector of dimension *K* with all zeros except for a 1 in position *k*. With this notation, the expression $E_{k}\delta \bar{\beta }$ denotes a vector of dimension *T* which contains the discrete-time derivatives of the *β*_*k*_(*t*) signal, that is the transmission rate for the *k*th class, and similarly $E_{k}\delta \bar{\gamma }$, $E_{k}\delta \bar{\nu }$ denote the vector of discrete-time derivatives of the recovery and death rate for the *k*th class.

The way in which we pursue sparsity in the derivatives of the parameters’ signals is by introducing *ℓ*_1_-type penalty functions in the minimization objective. The objective function to be minimized therefore takes the form

$$\begin{array}{ccc} f(\omega ,\bar{\theta })\; & \;≐\; & \;\frac{1}{T}\sum_{t=0}^{T-1}w^{T-t}\sum_{i=1}^{K}{∥\Delta_{i}\left(t+1\right)-W\Phi_{i}\left(t,\omega \right)\theta \left(t\right)∥}_{2}^{2}+\\ & & \;+\sum_{k=1}^{K}\;\lambda_{\beta }^{k}∥E_{k}\delta \bar{\beta }{∥}_{1}+\;\lambda_{\gamma }^{k}∥E_{k}\delta \bar{\gamma }{∥}_{1}\;+\;\lambda_{\nu }^{k}{∥E_{k}\delta \bar{\nu }∥}_{1}, \end{array}$$ (11)

where ${\bar{\theta }}^{⊤}≐\left[{\bar{\beta }}^{⊤}\;{\bar{\gamma }}^{⊤}\;{\bar{\nu }}^{⊤}\right]$, *W* is a diagonal weight matrix, and $\lambda_{\beta }^{k},\lambda_{\gamma }^{k},\lambda_{\nu }^{k}\geq 0$, *k* = 1, …, *K*, are tunable tradeoff parameters. Increasing the value of, say, $\lambda_{\beta }^{k}$ will tend to increase the sparsity of the discrete-time derivative of *β*_*k*_(*t*), and hence to reduce the number of different periods where *β*_*k*_(*t*) is constant. The estimation problem amounts to solving ${\text{min}}_{\omega ,\bar{\theta }\geq 0}f\left(\omega ,\theta \right)$ under constraints that *ω* ∈ [0, 1], and that *S*_*i*_(*t*)≥0 for all *t* = 0, 1, …, *T* and *i* = 1, …, *K*. The process is summarized in the following algorithm, which follows the same lines as Algorithm 1:

**Algorithm 2** (**Estimation of time-varying parameters**)

1. Assume suitable values of $\lambda_{\beta }^{k},\lambda_{\gamma }^{k},\lambda_{\nu }^{k}\geq 0$, *k* = 1, …, *K* are given; see comment below for a discussion on how to select them.
2. Grid *n* values *ω*_*i*_ of *ω* in [*ω*_min_, 1]. For each of these *ω*_*i*_:
3. Solve the constrained LASSO-type problem $f_{i}^{*}={\text{min}}_{\bar{\theta }\geq 0}f\left(\omega_{i};\bar{\theta }\right)$, with *f* given in (11), and let ${\bar{\theta }}_{i}^{*}$ be an optimal solution.
4. Repeat point 3 until the loop on *ω*_*i*_ is finished.
5. Let ω* be equal to the *ω*_*i*_ value that yielded the minimal value of $f_{i}^{*}$ in the inner iterations, and return the corresponding optimal parameter value ${\bar{\theta }}^{*}$.

The choice of the most appropriate values of $\lambda_{\beta }^{k},\lambda_{\gamma }^{k},\lambda_{\nu }^{k}\geq 0$, *k* = 1, …, *K* is done via repeated application of Algorithm 2 and ad-hoc adjustments. One suggestion is to start from common value for all classes, that is $\lambda_{\beta }^{k}=\lambda_{\beta }$, $\lambda_{\gamma }^{k}=\lambda_{\gamma }$, $\lambda_{\nu }^{k}=\lambda_{\nu }$, for all *k* = 1, …, *K*. Further, set relatively high values for λ_*γ*_ and λ_*ν*_, so that their corresponding signals result constant over the whole time horizon, and adjust the value of λ_*β*_ until a sensible number of periods (i.e., of constant “pieces” in the signal) is obtained. Constant pieces in the *β* signal correspond to periods in the pandemics evolution in which the containment measures are stable, such as the time in between of a lockdown enforcement date and its release date, and hence the total number of periods to be expected from the identified signal can reasonably be assessed a priori. Once a good λ_*β*_ value is obtained, we can proceed similarly by reducing the value of λ_*γ*_ and λ_*ν*_, always keeping in sight the total number of periods of the corresponding signals, which should be kept low. Finally, one can see if meaningful fitting improvements can be obtained by differentiating the λ values among classes.

## COVID-19 contagion in Italy

In this section we apply the proposed model with time-varying parameters to the various stages of COVID-19 contagion in Italy. We first present the dataset used for our analysis and then describe the proposed model fitting procedure.

### Italian COVID-19 data stratified by age class

In this analysis we consider the dataset presented in [11]. This dataset contains daily data about COVID-19 cases that occurred in Italy over the period Jan. 28, 2020 to March 20, 2021, divided into ten age classes of the population: 0-9 years, 10-19 years, 20-29 years, 30-39 years, 40-49 years, 50-59 years, 60-69 years, 70-79 years, 80-89 years and over 90 years. In our analysis we focused on the period of time from March 1, 2020 to March 20, 2021. We decided to discard data collected in January and February 2020, since in that period the data collection was partial, not regular and, thus, unreliable. Moreover, as observed in [11], this dataset is affected by a strong presence of noise and a high daily variability. For example, the number of new cases drops during weekends due to a reduced number of swab tests performed in weekends. In order to reduce such issues, we preprocess the data by computing the 7-day moving average. In addition to this dataset, we also use the data provided by the National Institute of Statistics (ISTAT) on the numerosity of the Italian population in each age class.

### Model fitting

**Fig 2:**
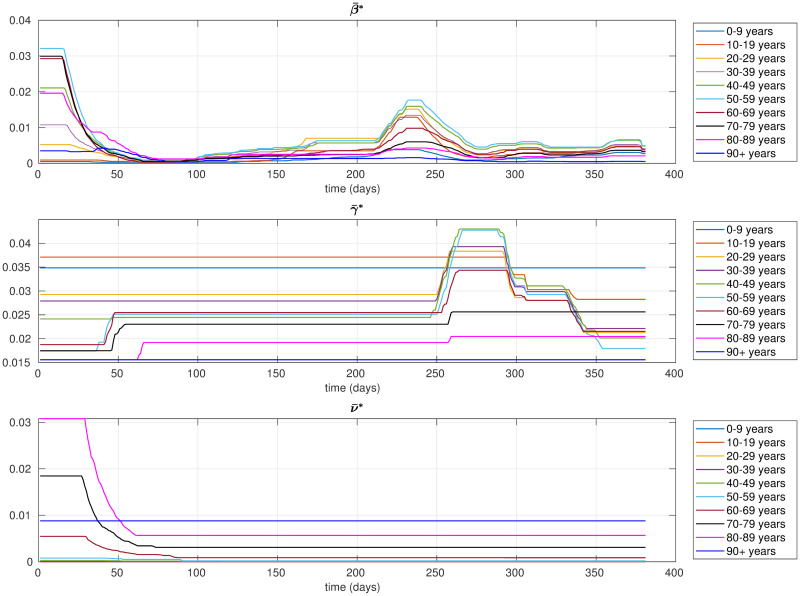
Optimal parameters identified by the proposed model. The percentage of non-zero elements in $E_{k}\delta {\bar{\beta }}^{*}$ is 44%, the percentage of non-zero elements in $E_{k}\delta {\bar{\gamma }}^{*}$ is 3%, and the percentage of non-zero elements in $E_{k}\delta {\bar{\nu }}^{*}$ is 10%.

To describe the contagion evolution of the various age classes, we considered the model with time-varying parameters described in previous section and we used the technique illustrated in Algorithm 2 to identify the optimal parameters of the model. For the estimation of the optimal parameters, we considered as training period the entire observation period from March 1, 2020 to March 20, 2021. As described in Algorithm 2, we first have to select suitable values of $\lambda_{\beta }^{k}$, $\lambda_{\gamma }^{k}$, $\lambda_{\nu }^{k}$ and then we compute the objective cost *f*(*ω*, ***θ***) as a function of *ω*. Since the contagion rates ***β*** are the parameters most subject to fluctuations over time, we choose to set higher values for $\lambda_{\gamma }^{k}$, $\lambda_{\nu }^{k}$ than $\lambda_{\beta }^{k}$. We set $\lambda_{\beta }^{k}=\lambda_{\beta }=7\times 10^{4}$, $\lambda_{\gamma }^{k}=\lambda_{\gamma }=2\times 10^{5}$ and $\lambda_{\nu }^{k}=\lambda_{\nu }=2\times 10^{5}$. Using these values, we obtained that the optimal value of *ω* is 1. Fig 2 shows the corresponding optimal parameters ${\bar{\theta }}^{{*}^{⊤}}=\left[{\bar{\beta }}^{{*}^{⊤}}\;{\bar{\gamma }}^{{*}^{⊤}}\;{\bar{\nu }}^{{*}^{⊤}}\right]$.

**Fig 3:**
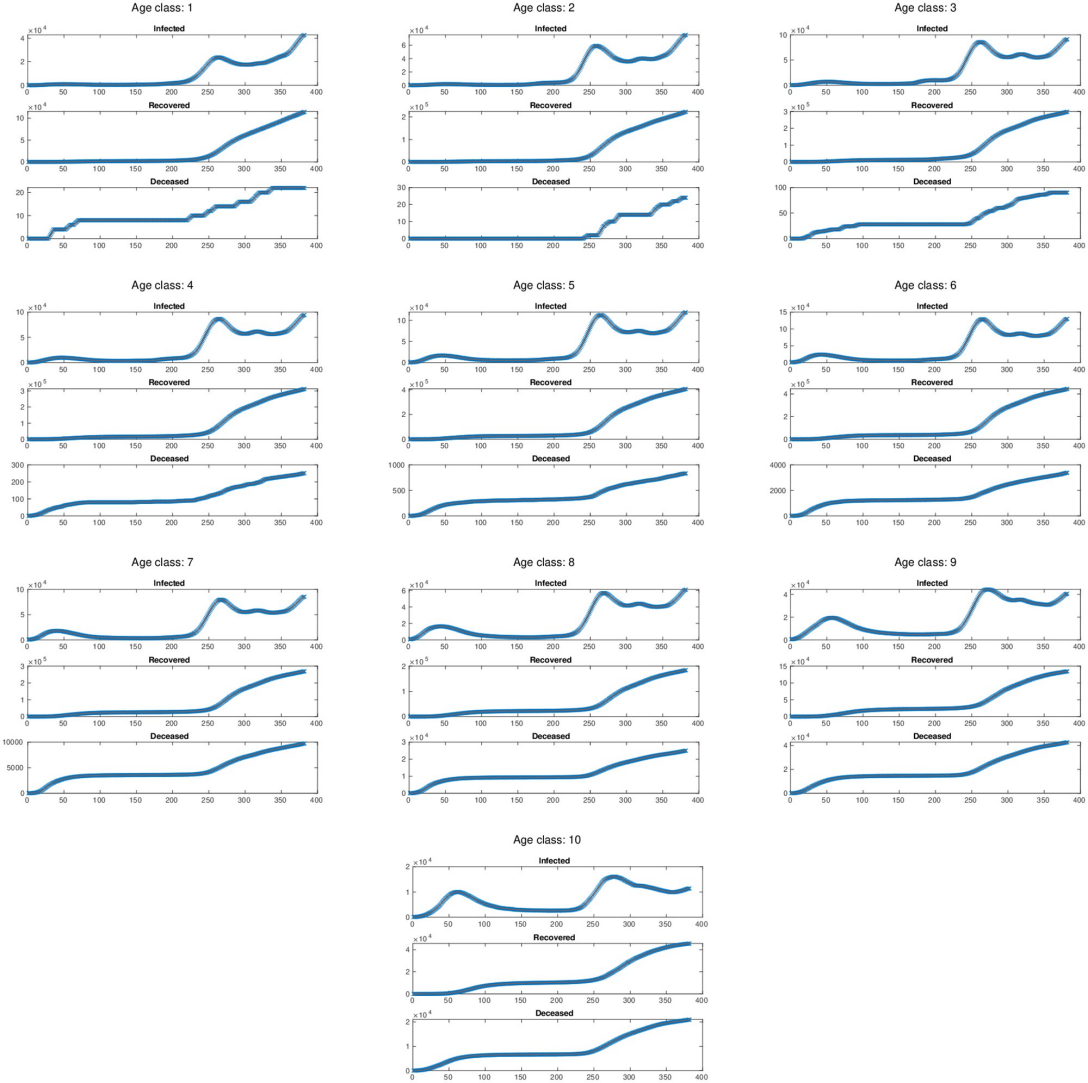
Per-class multi-simulation prediction using the optimal parameters ${\bar{\theta }}^{*}$. The red curve describes the multi-simulation prediction, and the blue crosses represent the actual data used for model training.

After having identified the parameters of the model, we can use the multi-simulation method described in [23] (Sec. 4.2) to obtain the prediction of the model using the estimated parameters. This method computes the state prediction *x*(*t*) at time *t* by means of an exponentially-weighted average of the predictions *x*(*t*, *t*_0_) obtained when the model starts at given initial conditions at *t*_0_ = 1, …, *T*, where 1 denotes the first day of our estimation period and *T* the last one. Fig 3 shows the resulting per-class prediction. We can observe that the predictions nicely fit the actual data for all the age classes. In order to quantitatively evaluate of the estimated model, we compute the root mean square error (RMSE) between the actual data and the model prediction. The results are reported in Table 1, which shows that the prediction error can be considered negligible.

**Table 1:** RMSE between actual data and model prediction for the infected, deceased and recovered curves.

Class	Infected	Recovered	Deceased
0-9 years	13	55	0
10-19 years	20	83	0
20-29 years	25	70	0
30-39 years	19	69	0
40-49 years	19	61	0
50-59 years	19	61	2
60-69 years	19	75	4
70-79 years	19	82	10
80-89 years	16	53	14
90 + years	3	21	10

## Contagion control by focused restrictions

**Fig 4:**
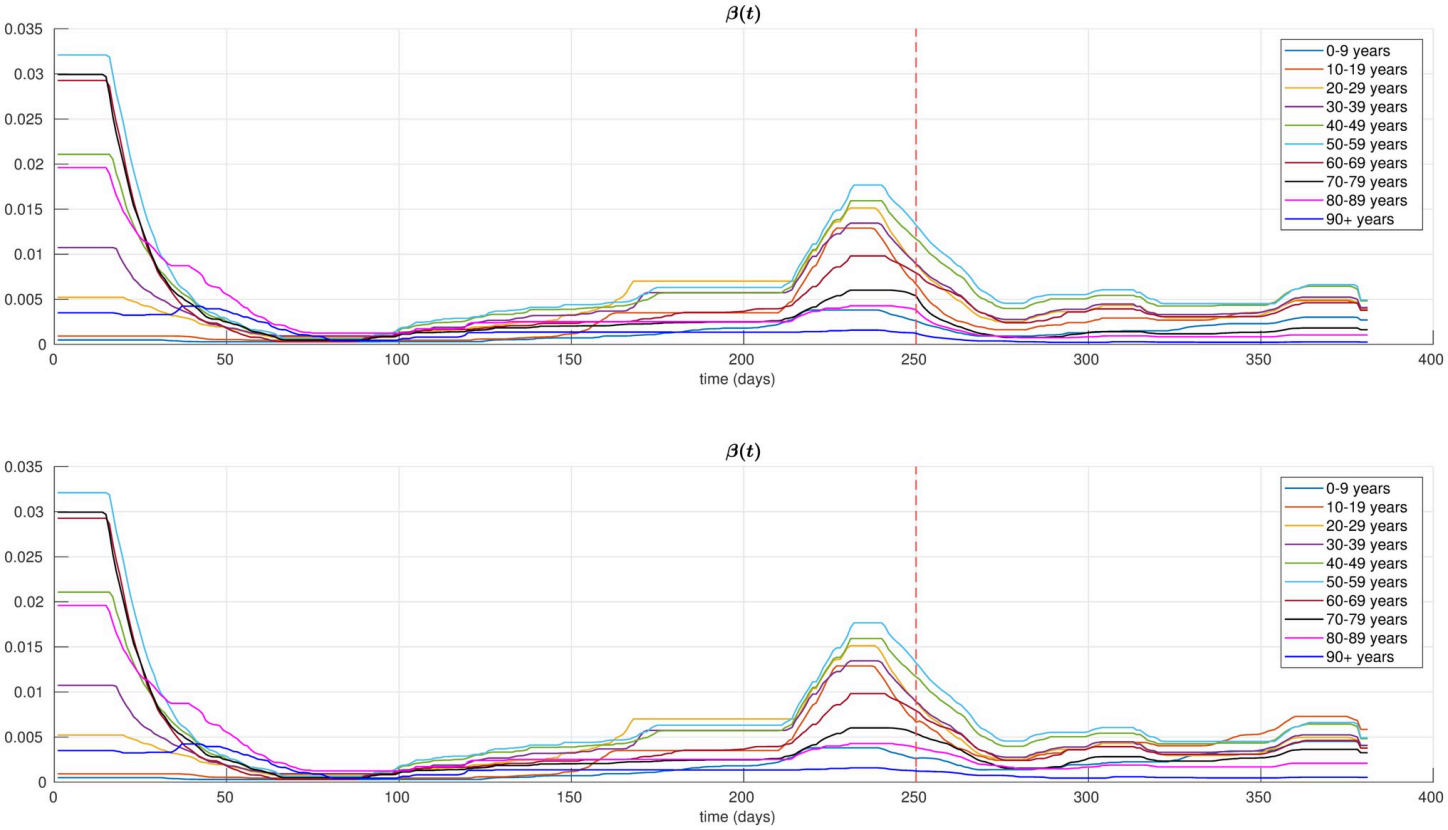
Time-varying contagion rates *β*_*i*_(*t*) in the two scenarios considered in the experimental section. A strengthened lockdown for the eldest age classes (top), a relaxed lockdown for the youngest age classes (bottom). The dashed red line denotes the start day of these scenarios.

**Fig 5:**
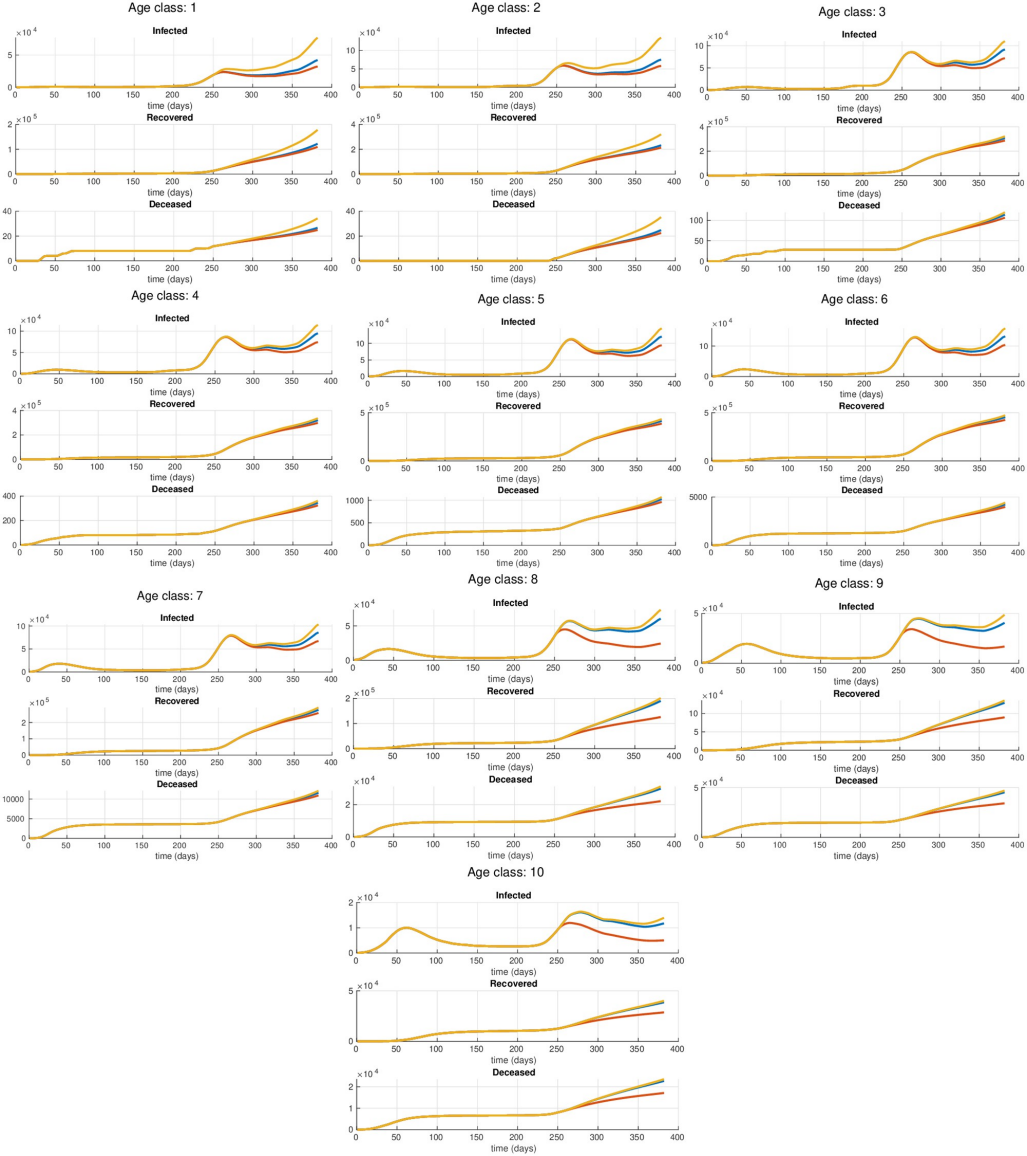
Per-class multi-simulation prediction. The blue curve corresponds to the case of a uniform lockdown obtained using the parameters ${\bar{\theta }}^{*}$ estimated from the actual data, the red curve corresponds to the case of a strengthened lockdown for the eldest age classes, and the yellow curve corresponds to the case of a relaxed lockdown for the youngest age classes. The selective restrictions start at day 250.

**Fig 6:**
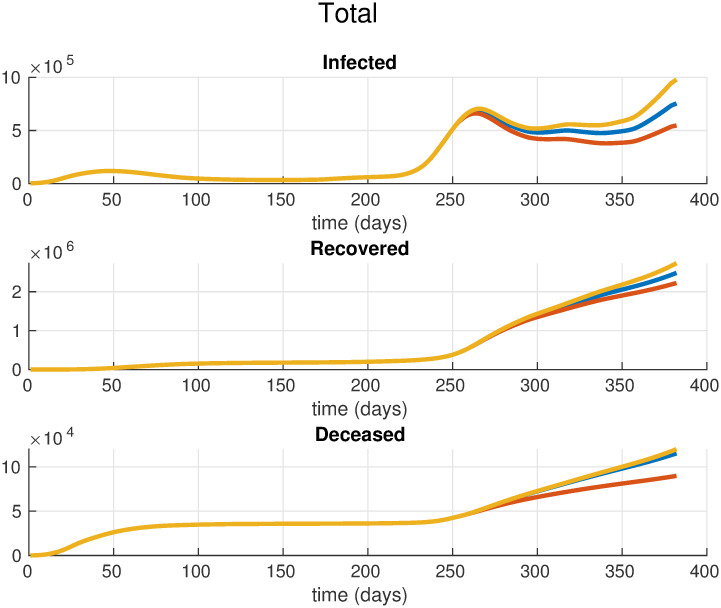
Multi-simulation prediction of the overall population. The blue curve corresponds to the case of a uniform lockdown obtained using the parameters ${\bar{\theta }}^{*}$ estimated from the actual data, the red curve corresponds to the case of a strengthened lockdown for the eldest age classes, and the yellow curve corresponds to the case of a relaxed lockdown for the youngest age classes. The selective restrictions start at day 250.

In this section, we are interested in analyzing the effects on the contagion evolution of focused restrictions that act only on specific age groups. In order to simulate the effects of such heterogenous lockdown policies, we modify the contagion rates *β*_*i*_(*t*) corresponding to a selected subset of age classes and evaluate the effects on the contagion evolution. For this analysis, we considered the time period from Nov. 5, 2020 to March 20, 2021. This time period corresponds to the introduction of lockdown policies in most of the italian regions, resulting in the closure of schools, recreational facilities and shops in most of the country. In particular, we consider two scenarios: a strengthened lockdown for the eldest age classes (i.e., over 70 years), or more relaxed restrictions for the population of schooling age (i.e., under 20 years). The second scenario is intended to investigate the effects of the school openings. For both cases, when *t* corresponds to the time period from Nov. 5, 2020 to March 20, 2021 we set the contagion rates $\beta_{i}\left(t\right)=\alpha_{i}\left(t\right){\bar{\beta }}_{i}^{*}\left(t\right)$, where ${\bar{\beta }}_{i}^{*}\left(t\right)$ is the optimal value estimated as described in the previous section and *α*(*t*) is a tuning factor. In the first scenario, we set *α*(*t*) as follows

$$\alpha_{i}\left(t\right)\;=\;\{\begin{array}{cc} 1\;-\;\phi +\;\text{exp}(-\frac{t-\tau }{\sigma }\;+\;\text{ln}\left(\phi \right)), & \;\text{if}\;i\geq 8\;\text{and}\;t\geq \tau \\ 1, & \;\text{otherwise} \end{array}\;,$$

where *τ* corresponds to the day when the restrictions were introduced (i.e., Nov. 5, 2020), *ϕ* is the reduction factor, and *σ* is a scaling parameter. In the experiments, we set *ϕ* = 0.5 and *σ* = 5. After a short transitory period, this definition of *α*(*t*) results in a reduction of 50% in the contagion rates of the eldest age classes. Instead, in the second scenario we set *α*(*t*) as follows

$$\alpha_{i}\left(t\right)\;=\;\{\begin{array}{cc} 1\;-\;\psi +\text{exp}(-\frac{t-\tau }{\sigma }+\text{ln}\left(\psi \right)), & \;\text{if}\;i\leq 2\;\text{and}\;t\geq \tau \\ 1, & \;\text{otherwise} \end{array},$$

where *ψ* is the increasing factor, and *σ* is a scaling parameter. In the experiments, we set *ψ* = 0.5 and *σ* = 5. Using this setting, after a short transitory period, we obtain an increment of 50% in the contagion rates of the youngest age classes. All the other parameters of the model are set equal to the optimal values of the model estimated in the previous section. Fig 4 shows the resulting parameters *β*_*i*_(*t*) as function of time. We now use the multi-simulation technique described in [23] to project forward in time these scenarios. In order to evaluate the effects of such type of focused restrictions, we compare these predictions against the multi-simulation prediction obtained using the optimal parameters ${\bar{\theta }}^{*}$ estimated in the previous section. This corresponds to the case where the restrictions are applied uniformly to the whole population. It is important to notice that, differently from what we have done in the previous section where the exponential average is computed using the entire observation period from March 1, 2020 to March 20, 2021, in this case we apply the multi-simulation method performing an exponential average of the predictions *x*(*t*, *t*_0_) with *t*_0_ = 1, …, *τ*, where *τ* corresponds to the start day of the considered scenarios (i.e., Nov. 5, 2020). Fig 5 depicts the resulting per-class multi-simulation predictions, instead Fig 6 shows the multi-simulation prediction of the overall population. In the case of a strengthened lockdown applied to the eldest age classes we can observe that, even though the restrictions are reinforced only for three classes, the effects are visible in all the age classes, significantly reducing the spread of the contagion and the overall number of deaths. Moreover, in Table 2 we compare the cumulative number of deaths in the three scenarios for the time period from Nov. 5, 2020 to March 20, 2021. In particular, it is important to observe that a selective lockdown applied only to the eldest age groups can result in a significant reduction of the total number of deaths, with a decrease of 35% with respect to the case of a uniform lockdown. Contrary, the results in Table 2 show that relaxing the restrictions for the youngest age classes may result in only a slight increase in the number of total deaths with respect to the uniform lockdown. On the one hand such increase, of the order of 6%, may be reasonably considered to be within the prediction error band of the model and, on the other hand in practice it could be easily offset by the introduction of other light non-pharmaceutical interventions on the released classes.

**Table 2:** Per-class cumulative number of deaths for the time period from Nov. 5, 2020 to March 20, 2021.

Class	Actual data	Uniform lockdown	Strengthened lockdown (class 8, 9, 10)	Relaxed lockdown (class 1, 2)
0-9 years	10	15	13	22
10-19 years	22	23	20	33
20-29 years	58	82	75	88
30-39 years	136	230	208	246
40-49 years	461	650	589	697
50-59 years	1909	2750	2500	2939
60-69 years	5599	7419	6744	7933
70-79 years	14199	19215	11434	20555
80-89 years	25217	27727	16700	29453
90 + years	12872	14715	9072	15516
Total	60483	72826	47355	77482

## Conclusions

In this paper we presented a modified SIRD model with age classes for the COVID-19 infection evolution. This model allows for introduction and analysis of control policies based on age-selective restrictions. In particular, we studied two scenarios: in the first one we considered strengthened restrictions for the eldest age classes, while in the second one we considered relaxed restrictions for the population of schooling age. The simulations suggest that such selective measures may provide a reduction of the mortality rate similar to the one obtained with a uniform lockdown, while having a lighter socio-economic impact.

In this work we focused on reducing the mortality rate. However, this is not the only quantity that needs to be controlled during the COVID-19 pandemic. There are other quantities that are important to monitor in order to analyze the evolution of the contagion, such as the hospitalisation or the Intensive Care Unit admission rates. In addition, the period of time considered in this work ends in March 2021, when the vaccination campaign was just beginning. For this reason, the proposed model does not take into account the vaccination rate. As future work, we might expand the proposed model in order to consider also these quantities.
